# Dataset of cannabis seeds for machine learning applications

**DOI:** 10.1016/j.dib.2023.108954

**Published:** 2023-02-07

**Authors:** Prawit Chumchu, Kailas Patil

**Affiliations:** a Kasetsart University, Sriracha, Thailand; b Vishwakarma University, Pune, India

**Keywords:** Cannabis detection, Cannabis seed image dataset, Computer vision, Deep learning, Machine learning, Seeds classification

## Abstract

The recent changes in policies in several countries regarding cannabis use has increased cannabis usage and research [1,2]. Cannabis is the second most used psychoactive substance word-wide [3]. Cannabis remains the subject of many research works. The cannabis can be classified into different classes according to their external features like colour, shape, and size using some computer vision and machine learning techniques. Precise classification or recognition is the unmet need of the agriculture business. This attracts many researchers to produce solutions with machine learning and deep learning techniques. Neat and clean dataset is the primary requirements to build accurate and robust machine learning model and minimize misclassification for the real-time environment. To achieve this objective, we have created an image dataset of cannabis seed. Accordingly, we have considered seventeen cannabis seeds to create dataset. The dataset contains 17 subfolders of cannabis seeds and folder is named with the category of seed. We strongly believe the cannabis seeds dataset will be very helpful for training, testing, and validation of cannabis classification or recognition with machine learning models.

**Specifications Table**

**Table:** 

Subject	Machine Learning, Agriculture Science
Specific subject area	Images dataset of cannabis seeds for classification
Type of data	Cannabis seeds images
How data were acquired	The high-quality cannabis seeds images were captured using mobile phone camera with different background and artificial lights.
Data format	Raw
Description of data collection	The high-resolution rear camera of iPhone was used to capture the different classes of cannabis seeds. The images were taken jpg. Format with the dimension of 3024 * 4032. The dataset is categorized into 17 subfolders of cannabis seeds namely Ak47 photo, blackberry (auto), cherry pie, gelato, gorilla purple, hang kra rog ku, hang kra rog phu phan st1, hang suea sakon Nakhon tt1, kd, kd_kt, krerng ka via, purple duck, skunk (auto), sour diesel (auto), tanaosri kan Daeng rd1, tanaosri kan kaw wa1, and thaistick foi thong. The images were taken at the white backgrounds. The proposed dataset can be used for training, testing and validation of cannabis seeds classification or reorganization with machine learning models.
Data source location	**KASETSART UNIVERSITY** Address: 199 Moo 6, Thung Sukla Subdistrict, Si Racha District, Chonburi Province 20230 Thailand. Latitude: 13° 7′ 11.02999″ N, Longitude: 100° 55′ 13.8900″ E Attitude: 45.41835
Data accessibility	Repository name: Dataset of Cannabis Seeds Data identification number doi:10.17632/dscww8w8zt.2. Direct URL to data: https://data.mendeley.com/datasets/dscww8w8zt

**Value of the Data**

• The dataset consists of 3434 high-quality original images of seventeen different classes of cannabis seeds.
• This is the first open access dataset to the best of our knolwege, of cannabis seeds.
• This dataset is useful to build applications of cannabis seeds classification, counting and detection with quality.
• The dataset will be useful to researcher to train, test and validate their classification or recognition machine learning models for cannabis seeds.
• The dataset is useful to build high quality cannabis seeds classification applications which are beneficial for farmers, agriculture industries, wholesalers, and cannabis seeds export companies.

## 1 Objectives

• A dataset of different types of cannabis seed that can help AI/ML algorithms to detect/classify cannabis seeds in real-time.
• A neat and clean dataset of cannabis seeds to build AI/ML models and minimize the misclassification by algorithms.

## 2 Data Description

**Fig. 1:**
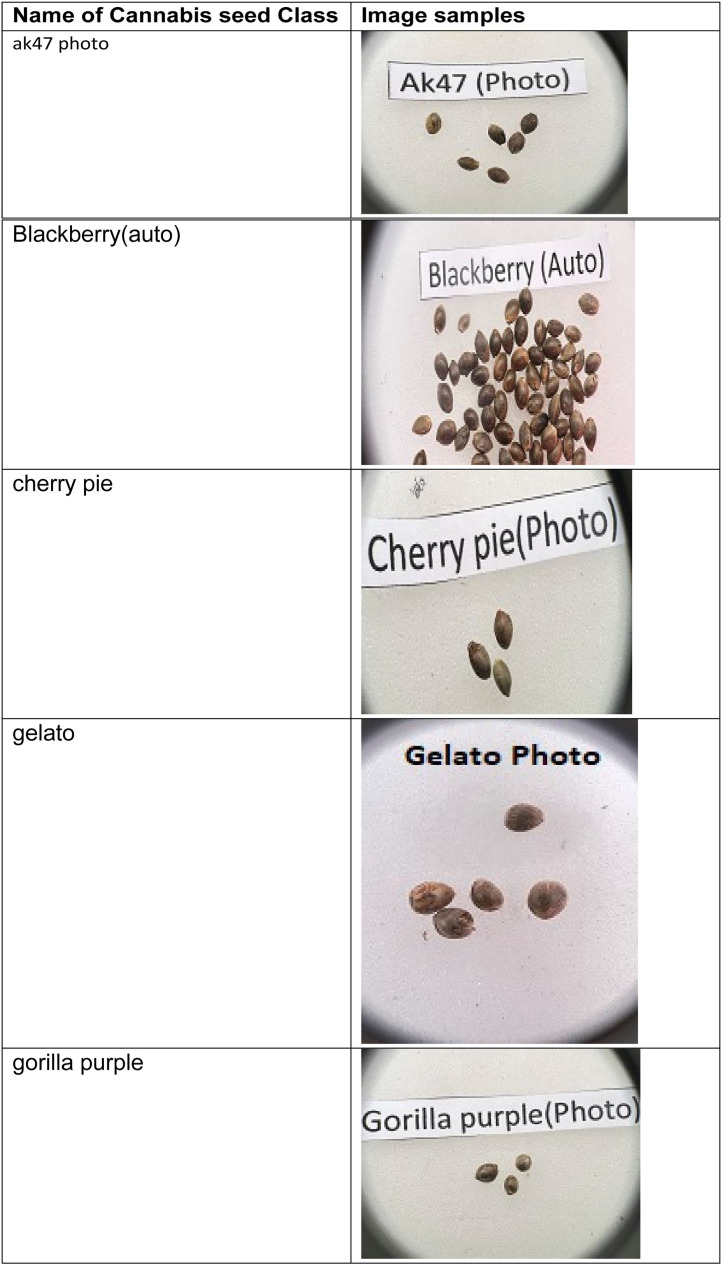

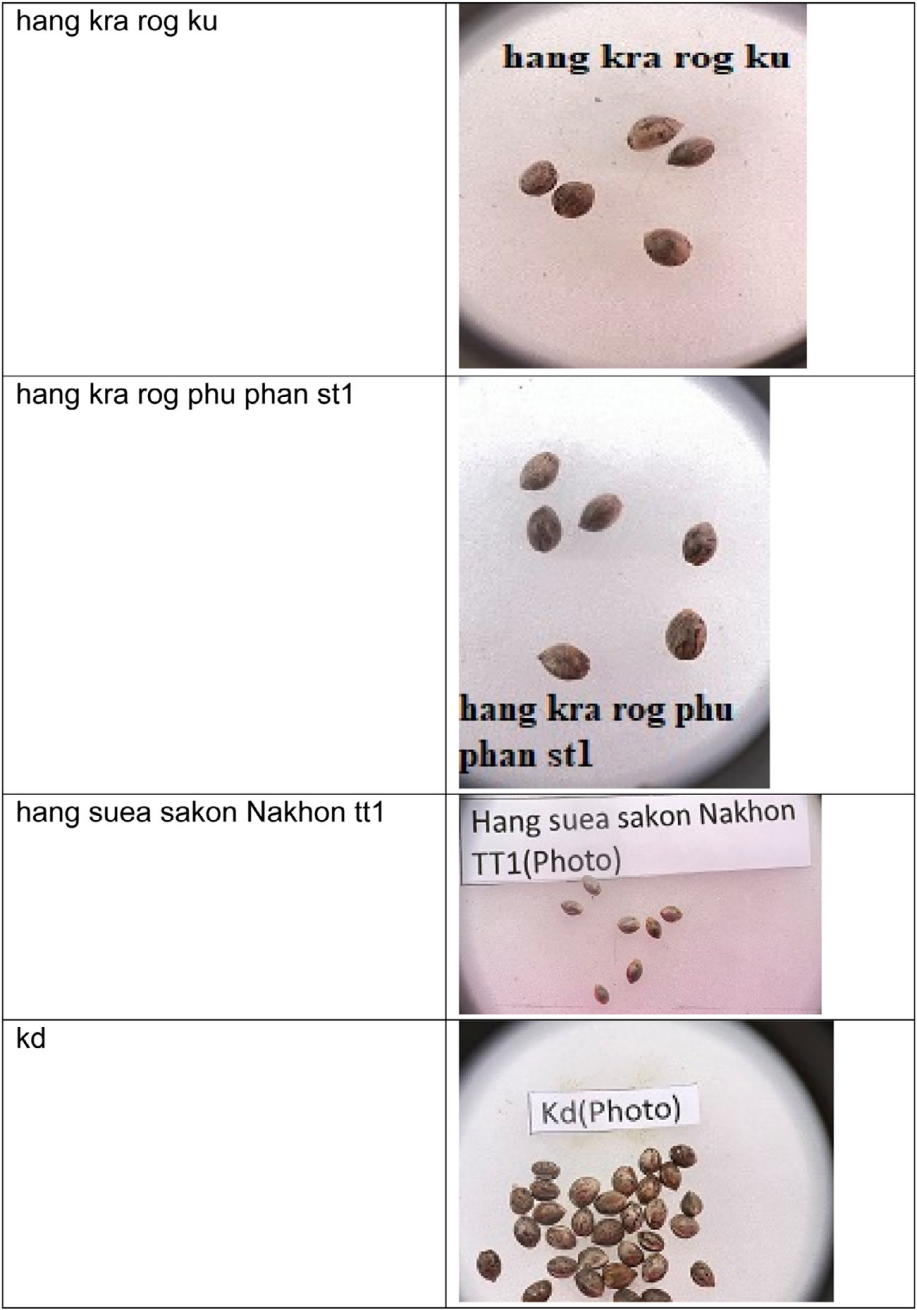

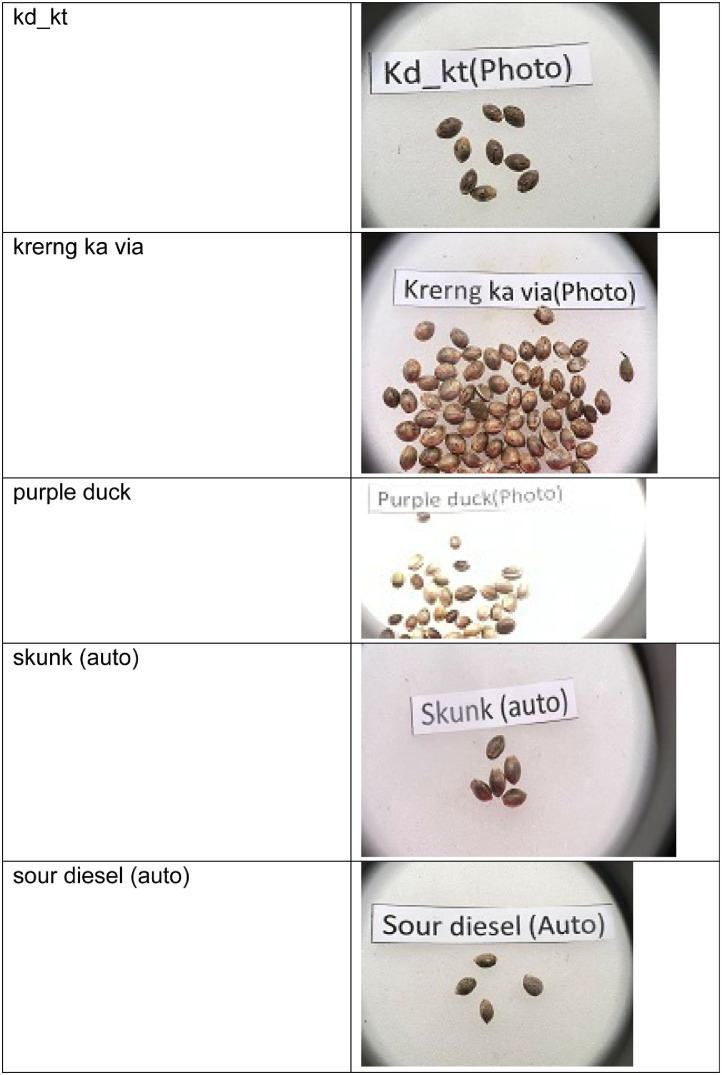

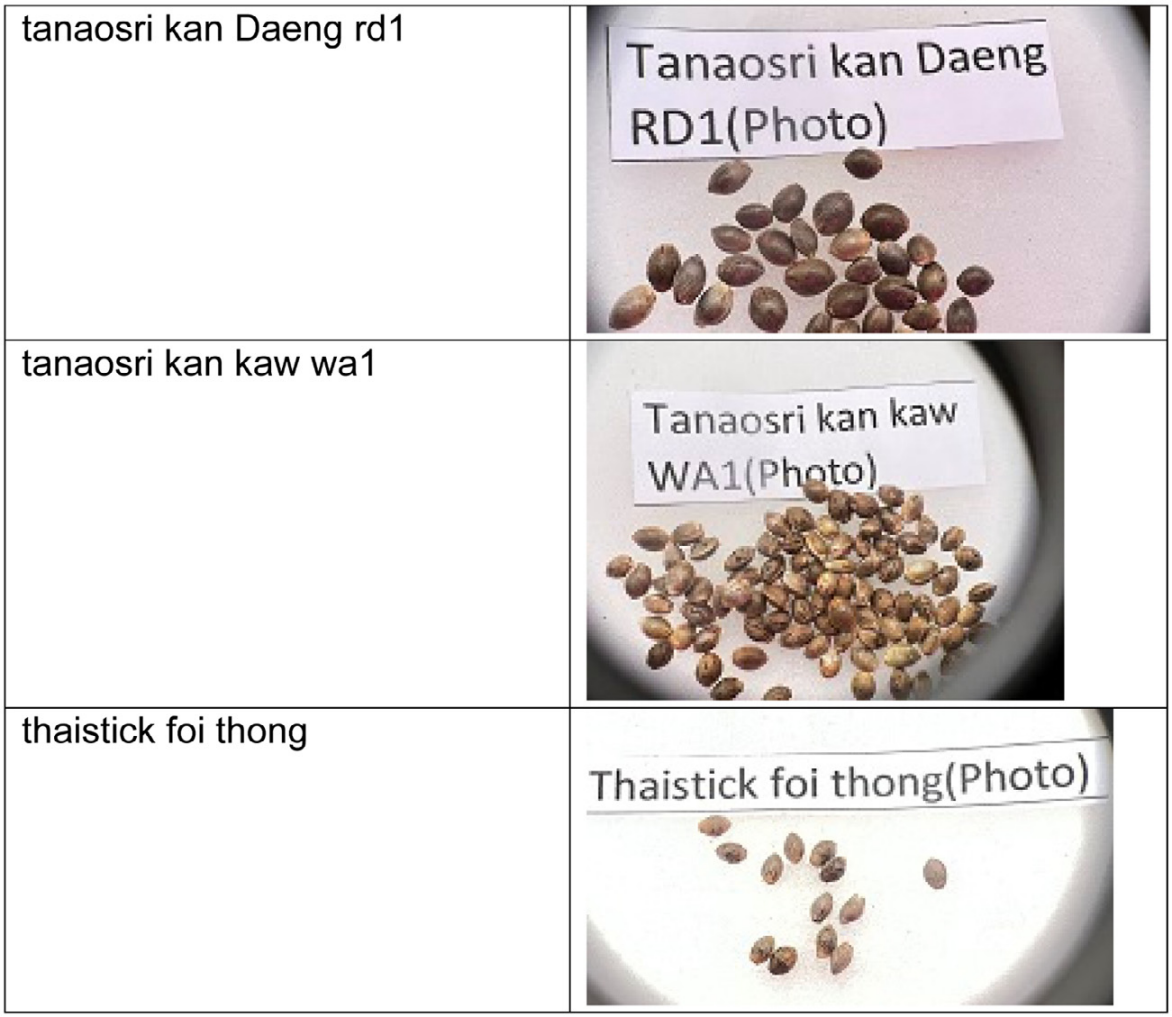
Cannabis seeds images for various categories

This dataset consists of seventeen classes of cannabis seeds namely Ak47 photo, blackberry (auto), cherry pie, gelato, gorilla purple, hang kra rog ku, hang kra rog phu phan st1, hang suea sakon Nakhon tt1, kd, kd_kt, krerng ka via, purple duck, skunk (auto), sour diesel (auto), tanaosri kan Daeng rd1, tanaosri kan kaw wa1, and thaistick foi thong. According to [4] cannabis seeds contain approximately 29 to 34 percent oil by weight. Cannabis seeds are also used to produce a clear yellow liquid. There is multiple usage of cannabis such as they can used for cosmetic preparations such as skin care products in the form of moisturizers, shampoos, lotions and lip balms. Cannabis seed oil is used as an ingredient in body oils and lipid-enriched creams[4]. There are multiple datasets on fruits, vegetables [5,^6^,7] but there is a need of cannabis seed dataset for researchers to develop machine learning models and/or applications. This dataset contains the images of cannabis seeds and not their plants’ leaves. The cannabis is cultivated in indoor and/or outdoor environments. The images were captured using mobile phone. The cannabis seed images were taken on white background. The Fig. 1 shows the sample images in the dataset consisting of images from each class.

## 3 Experimental Design, Materials and Methods

### 3.1 Experimental Design

**Fig. 2:**
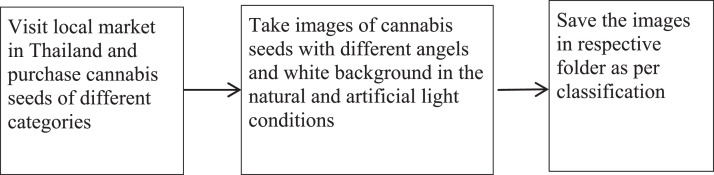
Canabis seed data acquisition Process

The image data acquisition process is shown in Fig. 2. The seed images were acquired using iPhone 13 pro mobile phone's high resolution rear camera. In all 3434 images were captured using camera and then were segregated and saved in respective folders.

The data acquisition process steps are shown in Table 1. The seed images are captured in the natural and artificial lighting conditions with different angles and background in months of June to October. Images are stored in original format in the dataset. Researchers can convert them into 256*256 or 224*224 as per their needs to build machine learning models with the cannabis seed dataset.

**Table 1:** Data acquisition steps.

Sr. No.	Step	Duration	Activity
1.	Data Gathering	June to October 2022	Purchase of Cannabis seed in Thailand for dataset. Daily captured the cannabis seed images in the natural and artificial light with different angles and white background.
2.	Pre-processing and creating dataset	October 2022	Save the images into respective folders as per their classification.

### 3.2 Materials or specification of image acquisition system

The seed images are captured using Apple iPhone 13 Pro of 12MP+12MP+12MP megapixels triple rear camera. All dataset images of original size 3024 × 4032. The images are stored in .jpg image format. The images acquired in variety of environmental conditions such as different light conditions, white background, and from different angles. The specifications of devices used for image acquisition and acquired images specifications are shown in Table 2 and 3 respectively.

**Table 2:** Specification of image acquisition device.

Sr. No.	Camera Particulars	Details
1	Camera maker	Apple
2	Camera Model	iPhone 13 Pro
3	F-stop	f/1.8
4	Exposure time	1/50 sec.
5	ISO Speed	ISO-640
6	Exposure bias	+1 step
7	Focal length	2 mm
8	Metering mode	Pattern
9	Flash mode	No flash, Compulsory
10	35mm focal length	26

### 3.3 Method

All the seventeen types of cannbis seeds Ak47 photo, blackberry (auto), cherry pie, gelato, gorilla purple, hang kra rog ku, hang kra rog phu phan st1, hang suea sakon Nakhon tt1, kd, kd_kt, krerng ka via, purple duck, skunk (auto), sour diesel (auto), tanaosri kan Daeng rd1, tanaosri kan kaw wa1, and thaistick foi thong were purchased from local market in thailand. The seeds brought to Kasetsart University laboratory. Daily images are captured using iPhone 13 pro mobile make with a high resolution rear camera in different angles and white backgrounds. The images were taken everyday. Table 4 describes the classes, number of image taken and the environments in which images are taken.

**Table 3:** Specification of images.

Sr. No.	Particulars	Details as per Vegetable classes
1	Dimension	3024 × 4032
2	Width	3024 pixels
3	Height	4032 pixels
4	Horizontal Resolution	72 dpi
5	Vertical Resolution	72 dpi
6	Bit Depth	24
7	Resolution unit	2

**Table 4:** Cannabis seeds dataset details.

Cannabis Seed type	Number of Images
ak47 photo	106
blackberry (auto)	203
cherry pie	50
gelato	327
gorilla purple	554
hang kra rog ku	153
hang kra rog phu phan st1	249
hang suea sakon Nakhon tt1	192
kd	49
kd_kt	147
krerng ka via	141
purple duck	151
skunk (auto)	233
sour diesel (auto)	327
tanaosri kan Daeng rd1	157
tanaosri kan kaw wa1	183
thaistick foi thong	212

**Total**	**3434**

## Ethics Statement

The data is available in public. No ethics approval needed for this study. There is no conflict of interest.

## CRediT Author Statement

**Prawit Chumchu:** Purchase of Seeds in Thailand, Data Collection, Data Validation, Writing – review & editing. **Kailas Patil:** Conceptualization, Methodology, Writing – original draft, Writing – review & editing.

## Declaration of Competing Interest

The authors declare that they have no known competing financial interests or personal relationships that could have appeared to influence the work reported in this paper.

## Data Availability

Dataset of Cannabis Seeds (Original data) (Mendeley Data).

## References

[1] Peacock A., Leung J., Larney S., Colledge S., Hickman M., Rehm J. (2018). Global statistics on alcohol, tobacco and illicit drug use: 2017 status report. Addiction.

[2] United Nations Office on Drugs and Crime (2019).

[3] Bahji A., Stephenson C. (2019). International perspectives on the implications of cannabis legalization: a systematic review & thematic analysis. Int. J. Environ. Res. Public Health.

[4] Anwar F., Latif S., Ashraf M. (2006). Analytical characterization of hemp (cannabis sativa) seed oil from different agro-ecological zones of Pakistan. J. Am. Oil. Chem. Soc..

[5] Meshram V.A., Patil K. (2022). FruitNet: Indian fruits image dataset with quality for machine learning applications. Data Br.

[6] Suryawanshi Y., Patil K., Chumchu P. (2022). VegNet: Dataset of vegetable quality images for machine learning applications. Data Brief.

[7] Meshram V., Patil K., Chumchu P. (2022). Dataset of Indian and Thai banknotes with annotations. Data Brief.

